# Angiotensin II‐mediated hippocampal hypoperfusion and vascular dysfunction contribute to vascular cognitive impairment in aged hypertensive rats

**DOI:** 10.1002/alz.13491

**Published:** 2023-10-10

**Authors:** Olivia Gannon, Sarah M. Tremble, Conor McGinn, Ruby Guth, Nadia Scoppettone, Ryan D. Hunt, Kirtika Prakash, Abbie C. Johnson

**Affiliations:** ^1^ Department of Neurological Sciences University of Vermont Larner College of Medicine Burlington Vermont USA

**Keywords:** aging, angiotensin II, chronic hypertension, hippocampus, vascular cognitive impairment, vascular dementia

## Abstract

**INTRODUCTION:**

Chronic hypertension increases the risk of vascular cognitive impairment (VCI) by ∼60%; however, how hypertension affects the vasculature of the hippocampus remains unclear but could contribute to VCI.

**METHODS:**

Memory, hippocampal perfusion, and hippocampal arteriole (HA) function were investigated in male Wistar rats or spontaneously hypertensive rats (SHR) in early (4 to 5 months old), mid (8 to 9 months old), or late adulthood (14 to 15 months old). SHR in late adulthood were chronically treated with captopril (angiotensin converting enzyme inhibitor) or apocynin (antioxidant) to investigate the mechanisms by which hypertension contributes to VCI.

**RESULTS:**

Impaired memory in SHR in late adulthood was associated with HA endothelial dysfunction, hyperconstriction, and ∼50% reduction in hippocampal blood flow. Captopril, but not apocynin, improved HA function, restored perfusion, and rescued memory function in aged SHR.

**DISCUSSION:**

Hippocampal vascular dysfunction contributes to hypertension‐induced memory decline through angiotensin II signaling, highlighting the therapeutic potential of HAs in protecting neurocognitive health later in life.

**Highlights:**

- Vascular dysfunction in the hippocampus contributes to vascular cognitive impairment.
- Memory declines with age during chronic hypertension.
- Angiotensin II causes endothelial dysfunction in the hippocampus in hypertension.
- Angiotensin II‐mediated hippocampal arteriole dysfunction reduces blood flow.
- Vascular dysfunction in the hippocampus impairs perfusion and memory function.

## 1 BACKGROUND

It is estimated that over 55 million people currently live with dementia, and that number is projected to more than double by 2050.^1^ Current estimates report that one in 10 people over the age of 65 have dementia, a startling statistic that is only expected to rise given the aging population.^2^ Alzheimer's disease (AD) is the most common form of dementia.^2^ The second leading cause of age‐related dementia is vascular cognitive impairment (VCI), which includes a spectrum of cognitive dysfunction due to cerebrovascular disease, with the most severe form being vascular dementia.^2^, ^3^ Importantly, recent findings suggest that the majority of patients diagnosed with AD actually have mixed dementia, which includes the classic AD neurodegeneration as well as cerebrovascular pathology.^2^, ^4^, ^5^, ^6^ Therefore, the incidence of VCI is likely underestimated. While aging is the leading non‐modifiable risk factor for dementia, there are clear modifiable risk factors for VCI, such as chronic hypertension.^3^ Therefore, understanding how chronic hypertension and concomitant aging affect the cerebral microvasculature of brain regions central to memory and cognition is important to treat and, hopefully, prevent VCI.

The risk for VCI in people with chronic hypertension is up to 60% greater compared to normotensive individuals.^7^, ^8^, ^9^, ^10^ This is in part due to hypertension increasing the incidence of ischemic stroke and, therefore, post‐stroke dementia, which is a form of VCI; however, hypertension also directly affects brain regions central to learning and memory, including the hippocampus.^3^, ^11^, ^12^ The hippocampus is a bilateral, deep brain structure critical to many core aspects of memory and cognitive function and is more susceptible to hypoxic/ischemic injury compared to other brain regions.^13^ Hippocampal atrophy, a reduction in hippocampal volume, is widely known to be associated with cognitive impairment and occurs under several pathological conditions, including in individuals with chronic hypertension. In fact, high blood pressure predicted hippocampal atrophy rate in cognitively impaired individuals over 70 years old, and longer duration of hypertension was associated with lower hippocampal volume.^14^, ^15^, ^16^ A reduction of blood flow (hypoperfusion) to the hippocampus is considered a primary mechanism by which neuropathological changes occur during chronic hypertension, including hippocampal atrophy, and is a predictor of cognitive decline.^17^, ^18^ The hippocampal vasculature and vascular reserve are becoming recognized as important contributors to maintaining neurocognitive health and memory function later in life.^19^, ^20^ Therefore, it is important to understand how aging during chronic hypertension affects the vasculature that supplies the hippocampus and the relationship between hypertension‐induced hippocampal vascular dysfunction and changes in hippocampal perfusion and memory function.

In this study, we first investigated how age with concomitant hypertension affected memory function, hippocampal blood flow, and hippocampal arteriole (HA) function, including myogenic reactivity and vasoreactivity to mediators involved in neurovascular coupling (NVC). We used a rat model of VCI, spontaneously hypertensive rats (SHR), and studied hippocampal hemodynamics in early, mid, and late adulthood. We hypothesized that memory function would progressively decline with age due to hippocampal vascular dysfunction and reduced hippocampal perfusion. Secondly, we investigated the potential roles of angiotensin II (Ang II) signaling and oxidative stress as underlying mechanisms by which chronic hypertension may impair hippocampal vascular function by chronically treating SHR in late adulthood with the angiotensin converting enzyme (ACE) inhibitor captopril or the antioxidant apocynin. We hypothesized that inhibiting Ang II signaling during chronic hypertension would improve hippocampal vascular function, restore hippocampal blood flow, and protect memory function in late adulthood.

## 2 METHODS

### 2.1 Animals

Experiments were conducted using male Wistar rats (4 to 5 months old) or male SHR that were either 4 to 5 months (early adulthood), 8 to 9 months (mid‐adulthood), or 14 to 15 months of age (late adulthood) and were purchased from Charles River, Canada. To investigate the underlying mechanisms by which memory, hippocampal perfusion, and/or HA function may be impaired as a function of age during hypertension, SHR in late adulthood were treated with either captopril (+Capto; 100 mg/kg/day in drinking water for 3 months), apocynin (+Apo; 12 mg/kg/day in drinking water for 2 months), or were vehicle controls that received regular drinking water. The dose and duration of captopril treatment were based on a previous study showing attenuated hypertension‐induced structural remodeling of pial arterioles in 15‐month‐old SHR.^21^ For apocynin, dose and duration were based on our own study showing that this dose improved HA function in an experimental rat model of hypertensive pregnancy,^22^ and this duration is protective of cardiovascular health in SHR.^23^

To understand how hippocampal function is affected by age during chronic hypertension, three primary stages of adulthood were studied: early, mid, and late adulthood. Although healthy rats have a typical life span of ∼3 years, SHR begin to go into heart failure at 16 to 18 months of age.^24^ SHR that are 14 to 15 months old are no longer in the same phase of adulthood as normotensive rats and are considered to be older.^25^ Therefore, we did not include age‐matched normotensive rats in this study, as direct age‐matching may not be a valid comparison between rats with chronic hypertension and rats that are normotensive. Male rats were used because hypertension in older men is associated with greater hippocampal atrophy, and the incidence for vascular dementia has been reported to be higher in men than in women.^15^, ^26^, ^27^, ^28^ Adult SHR are a widely used rodent model to investigate the effect of chronic hypertension on cognitive decline and share a neuropathology of VCI similar to that which occurs in humans.^29^ Therefore, this model is useful in the investigation of how aging and chronic hypertension affect memory, hippocampal perfusion, and vascular function. Wistar‐Kyoto (WKY) rats are often used as the genetic control for SHR. However, Wistar rats were used as normotensive controls rather than WKY because WKY rats are an established model of depression and anxiety‐like behavior that could confound our investigation of hippocampal hemodynamics as it relates to memory.^30^, ^31^, ^32^

All rats were purchased at 4 months of age and allowed to age in the University of Vermont Animal Care Facility, an Association for Assessment and Accreditation of Laboratory Animal Care International‐accredited facility. Rats were housed in pairs with environmental enrichment, including a tunnel and a wooden chew block. Ambient temperature in the animal room where all the rats were housed was maintained within the guideline‐recommended 20°C to 26°C, and the rats were fed standard rat chow (LabDiet ProLab IsoPro RMH 3000, ScottPharma Solutions, Marlborough, MA, USA). Rats acclimated to the animal facility for at least 5 days prior to handling and were maintained on a 12‐h light/dark cycle and allowed access to food and water ad libitum. All procedures occurred at the same time of day and were approved by the Institutional Animal Care and Use Committee (IACUC) and conducted in accordance with the National Institutes of Health (NIH) Guide for the Care and Use of Laboratory Animals. Investigators were blinded to animal groups during behavioral and blood flow analyses. All euthanasia was under either isoflurane anesthesia or chloral hydrate anesthesia, according to NIH guidelines.

### 2.2 Assessment of learning and memory function

To understand how chronic hypertension and age impacted hippocampal‐dependent cognitive function and whether treatment could improve memory, behavioral tasks were used to assess long‐term memory and spatial working memory (*n* = 6 ‐ 8/group) the week prior to terminal experiments. We compared two types of memory to investigate the potential network‐specific effects of hypertension/age: long‐term memory is considered largely hippocampal‐dependent and was tested using a novel object recognition (NOR) task; spatial working memory is hippocampal‐prefrontal cortex‐dependent and was tested using a continuous Y maze task, as we have done previously.^33^, ^34^, ^35^, ^36^, ^37^ For the NOR task, rats habituated to the open field arena for 5 min, followed by a 10‐min acquisition period when the rats freely explored two identical objects.^38^ After a 48‐h consolidation period, rats were placed in the same arena containing one familiar object and one novel object. The time that each rat spent investigating the novel and familiar objects was measured and used to calculate a recognition index as a measure of long‐term memory function. For the continuous Y maze task, rats freely explored a Y maze for 8 min, and spontaneous alternation behavior and total arm entries were determined and used to calculate an alternation index as a measure of spatial working memory.^34^ All tasks were video recorded and videos analyzed by two independent reviewers that were blinded to experimental group.

**RESEARCH IN CONTEXT**

1. **Systematic review**: The authors reviewed the literature online, including published abstracts. The vasculature of the hippocampus is relatively understudied; however, a few recent publications highlight the importance of the vasculature of the hippocampus in cognitive decline associated with aging and cerebrovascular disease from chronic hypertension. These have been cited appropriately.
2. **Interpretation**: We provide evidence that protecting the hippocampal vasculature in chronic hypertension in late life improves hemodynamics and memory function. Our findings broaden our understanding of how chronic hypertension contributes to vascular cognitive impairment and of how anti‐hypertensive medications targeting angiotensin II signal slow memory decline.
3. **Future directions**: The manuscript provides a framework for novel hypotheses and continued investigation of the role of the hippocampal vasculature in dementia. Examples include further understanding (a) the causal role of impaired neurovascular coupling in the hippocampus in memory decline; (b) hippocampal vascular dysfunction during healthy aging in both sexes, including the post‐menopausal state; (c) other pathological states associated with memory loss, including Alzheimer's disease (AD), post‐stroke dementia, and psychiatric disorders.

### 2.3 Measurement of hippocampal perfusion

To investigate how hippocampal perfusion may change with age during chronic hypertension and its potential role in memory decline, absolute hippocampal blood flow was measured using a hydrogen clearance method, as we have done previously.^35^ Separate groups of rats (*n* = 6/group) were anesthetized initially with isoflurane (1% ‐ 3% in oxygen) for instrumentation. One femoral vein was cannulated for administration of the anesthetic chloral hydrate (50 mg/ml), and femoral arteries were cannulated for continuous blood pressure recordings via a pressure transducer (BIOPAC Systems Inc., Goleta, CA, USA) and to obtain blood samples for blood gas measurements. Rats were intubated and mechanically ventilated to maintain blood gases and pH within the normal physiological range and body temperature was maintained at 37.0 ± 0.2°C. After instrumentation, rats were placed in prone position, secured in a stereotaxic apparatus and slowly tapered off of isoflurane anesthesia, and maintained on chloral hydrate anesthesia for the remainder of the experiment. Chloral hydrate was used because it is less vasoactive than isoflurane and has been used in studies measuring hippocampal blood flow via the hydrogen clearance method.^35^, ^39^, ^40^, ^41^

The hydrogen microsensor was calibrated daily, and hydrogen current was sampled at 5 Hz and recorded using a multimeter (Unisense, Aarhus, Denmark). Through a 2‐mm burr hole, a glass hydrogen microsensor with a 50‐μm tip containing both the sensing anode and the reference electrode (Unisense, Aarhus, Denmark) was inserted into the CA1 region of the dorsal hippocampus. The CA1 region was studied because of its high susceptibility to ischemic injury and its prominent role in learning and memory.^42^, ^43^, ^44^ For rats in early and mid‐adulthood, coordinates were 3.5 mm posterior, 3.0 mm lateral, and 2.0 mm ventral from bregma.^35^, ^45^ For the SHR in late adulthood, coordinates were adjusted for decreased brain volume (as determined in pilot studies) and were 3.5 mm posterior, 2.8 mm lateral, and 2.0 mm ventral from bregma. After placement of the hydrogen microsensor, rats inhaled 4% hydrogen gas until the hydrogen current reached a steady state, at which point saturation was reached. Hydrogen gas was then turned off and tissue desaturation was recorded. The half‐life of hydrogen clearance was obtained from tissue desaturation measurements and used to calculate absolute blood flow, as we have previously done.^22^, ^35^, ^39^ One SHR in mid‐adulthood was excluded due to technical difficulties during H_2_ clearance measurements. One vehicle treated SHR and one apocynin‐treated SHR in late adulthood spontaneously died prior to experimentation.

### 2.4 Isolated hippocampal arteriole function

To understand the potential role of the hippocampal vasculature in hippocampal dysfunction occurring during hypertension and aging, HAs supplying the dorsal hippocampus were isolated and studied using pressure myography, as previously described.^35^, ^36^, ^39^ Briefly, rats that had previously undergone behavioral testing (*n* = 6 ‐ 8/group) were decapitated under isoflurane anesthesia (3% in oxygen) and brains immediately dissected and placed in cold, oxygenated artificial cerebrospinal fluid (aCSF). HAs were carefully dissected, mounted, and secured on glass cannulas with nylon monofilament and pressurized in an arteriograph chamber (Living Systems Instrumentation, Burlington, VT, USA).^35^, ^39^ HAs from normotensive and hypertensive rats were equilibrated for 1 h at 20 mmHg. Intravascular pressure was increased to 120 mmHg in a stepwise manner to determine whether arterioles developed spontaneous myogenic tone and to measure myogenic reactivity. Lumen diameter and vascular wall thickness were recorded at each intravascular pressure once stable. Pressure was then returned to 40 in HAs from Wistar rats or 60 mmHg for SHR for the remainder of the experiment.

To investigate endothelial function of HAs, specifically small‐ and intermediate‐conductance calcium‐activated potassium (SK_Ca_/IK_Ca_) channel function, vascular responses to pharmacological manipulation of SK_Ca_/IK_Ca_ channels were measured: the SK_Ca_/IK_Ca_ channel agonist NS309 (10^−8^ ‐ 10^−5^ M), the selective SK_Ca_ channel antagonist apamin (300 nM), and the selective IK_Ca_ channel antagonist TRAM‐34 (1 μM). To investigate the responses of HAs to mediators central to NVC in the hippocampus, reactivity to adenosine (10^−8^ ‐ 10^−5^ M) and the nitric oxide (NO) donor sodium nitroprusside (SNP; 10^−8^ ‐ 10^−5^ M) were measured. At the end of each experiment, aCSF was replaced with aCSF containing zero calcium, papaverine (10^−4^ M), and diltiazem (10^−5^ M) to fully relax the vascular smooth muscle and passive structural measurements made within the pressure range of 5 to 120 mmHg.

### 2.5 Non‐invasive blood pressure measurements

To determine any anti‐hypertensive effects of captopril and/or apocynin treatment in SHR in late adulthood, blood pressures were measured non‐invasively via the tail‐cuff method using the CODA 8 System (Kent Scientific, Torrington, CT, USA), as described previously.^46^, ^47^ Briefly, rats were allowed to acclimate to the room in their home cages for 1 h before measurements were taken. Rats were placed in individual tube holders, blood pressure tail‐cuffs were applied at the base of the tail, and the rats were kept warm with a heating pad. All rats underwent three training days to acclimate to the apparatus, and systolic and diastolic blood pressures were measured weekly for the duration of treatments and in age‐matched vehicle control SHR prior to behavioral testing and being euthanized for isolated vessel experiments (*n* = 6 to 8/group).

### 2.6 Drugs and solutions

Diltiazem was purchased from MP Biomedicals (Santa Ana, CA, USA), apamin and TRAM‐34 were purchased from Tocris Bioscience (Minneapolis, MN, USA), and captopril was purchased from Abcam (Cambridge, MA, USA). All other compounds, including apocynin, chloral hydrate, NS309, adenosine, SNP, papaverine, and those used to make aCSF, were purchased from Sigma‐Aldrich (St. Louis, MO, USA). Apocynin and captopril were diluted in drinking water based on the rats’ average weight for the previous week and the average amount of water drank daily, and were made fresh weekly. Apocynin required being dissolved in hot (∼60°C) tap water while mixing. Chloral hydrate was made daily in sterile lactated Ringer's solution. Stock solutions of adenosine, SNP, papaverine, and diltiazem were made weekly and stored at 4°C until use. NS309, apamin, and TRAM‐34 stock solutions were aliquoted and stored at −20°C until use. aCSF contained (mM) NaCl 122.0, NaHCO_3_ 26.0, NaH_3_PO_4_ 1.25, KCl 3.0, MgCl_2_ 1.0, and CaCl_2_ 2.0. Buffer solutions were made each week and stored without glucose at 4°C. Glucose was added (4.0 mM) immediately prior to each experiment. Zero‐calcium aCSF was made similarly, omitting the addition of CaCl_2_. The aCSF was aerated with 5% CO_2_, 10% O_2_, and 85% N_2_ to maintain pH at 7.40 ± 0.05, and the temperature within the arteriograph chamber was maintained at 37.0 ± 0.1°C throughout the experiments.

### 2.7 Data calculations

To determine memory function, recognition index was calculated using the following formula: Time_Novel Object_/Time_Total_, where Time_Novel_ is the amount of time (s) a rat spent investigating the novel object and Time_Total_ is the total time (s) that the rat spent investigating both objects. Locomotion was assessed by calculating average speed during the 5 min exploration of the open field arena (habituation phase of the novel object recognition task) using the following formula: total distance traveled (m)/300 s. The spontaneous alternation index was calculated using the following formula: [No. alternations/(No. arm entries − 2)]. Absolute hippocampal blood flow was calculated from the rate of hydrogen clearance using the initial slope to determine the first‐order clearance rate in which flow (mL/s) = 0.693/t_1/2_, where t_1/2_ is the time (s) to reach half of the maximal tissue concentration of hydrogen and then converted to mL/100 g tissue/min using following equation: Blood flow = (0.693 × 100 g × 60 s)/ t_1/2_.^48^

For isolated HA experiments, percent myogenic tone was calculated using the following equation: Percent tone = [1 − (φ_active_/φ_passive_)] × 100%, where φ_active_ is the lumen diameter under active conditions and φ_passive_ is the lumen diameter under fully relaxed conditions at a specific intravascular pressure. Percent reactivity to NS309, adenosine, and SNP was calculated from the following equation: Percent reactivity = [(φ_dose_ − φ_baseline_)/(φ_passive_ − φ_baseline_)] × 100%, where φ_dose_ is the lumen diameter of the vessel after treatment with a specific concentration of drug and φ_baseline_ is the starting lumen diameter before any drug treatment. To calculate the EC_50_ values of NS309, concentrations of NS309 were transformed to logarithmic and a non‐linear regression was performed using a three‐parameter model. EC_50_ was calculated from the equation, where Y = 50: Y = Bottom percentage reactivity + (100% reactivity − Bottom percentage reactivity)/(1+10^((LogEC50−X)^)). Percent constriction to apamin and subsequent TRAM‐34 was calculated as a percent change in lumen diameter from baseline by the following equation: Percent constriction = [1 − (φ_drug_/φ_baseline_)] × 100%, where ϕ_drug_ is the lumen diameter after drug exposure and ϕ_baseline_ is the diameter before giving the drug. The outer diameter (φ_outer_) was calculated at each pressure by the following equation: φ_outer_ = φ_inner_ + 2WT, where φ_inner_ is the inner (lumen) diameter of the vessel fully relaxed and WT is the measured wall thickness. Cross‐sectional area (CSA) was calculated by the following equation: CSA = π(φ_outer_/2)2 − π(φ_inner_/2)2 at 5 mmHg and 60 mmHg intravascular pressure. Percent distensibility was calculated using the following equation: Percent distensibility = [(φ_passive_ − φ_5_)/φ_5_] × 100%.

### 2.8 Statistical analyses

The number of animals used in each experiment was determined by a statistical power calculation using a two‐sided 95% confidence interval for a single mean and 1 – β of 0.80 based on previous studies using a similar methodology.^35^, ^39^ We determined that *n* = 6/group was sufficient to detect statistical differences in hippocampal perfusion, vasoreactivity of HAs to mediators of neurovascular coupling, and novel object recognition and Y maze tasks. All statistical testing was performed using GraphPad Prism 9.3 software (GraphPad Software Inc., La Jolla, CA, USA). Results are presented as mean ± SEM, and a *p* value of < 0.05 was considered significant. Differences between groups in memory, perfusion and vascular lumen diameters, myogenic tone, and vasoreactivity were determined using an ordinary one‐way analysis of variance (ANOVA) with a Tukey's post hoc test to correct for multiple comparisons. Differences in EC_50_ concentrations were determined using a non‐parametric Kruskal‐Wallis ANOVA with a post hoc Dunn's test to correct for multiple comparisons. Rats were randomized to treatment group, and experiments and analyses were completed in a blinded fashion whenever possible.

### 2.9 Data availability

Data will be made available upon reasonable request.

## 3 RESULTS

### 3.1 Effects of age on memory and hippocampal hemodynamics during chronic hypertension

#### 3.1.1 Long‐term memory progressively declined with age during chronic hypertension

**FIGURE 1:**
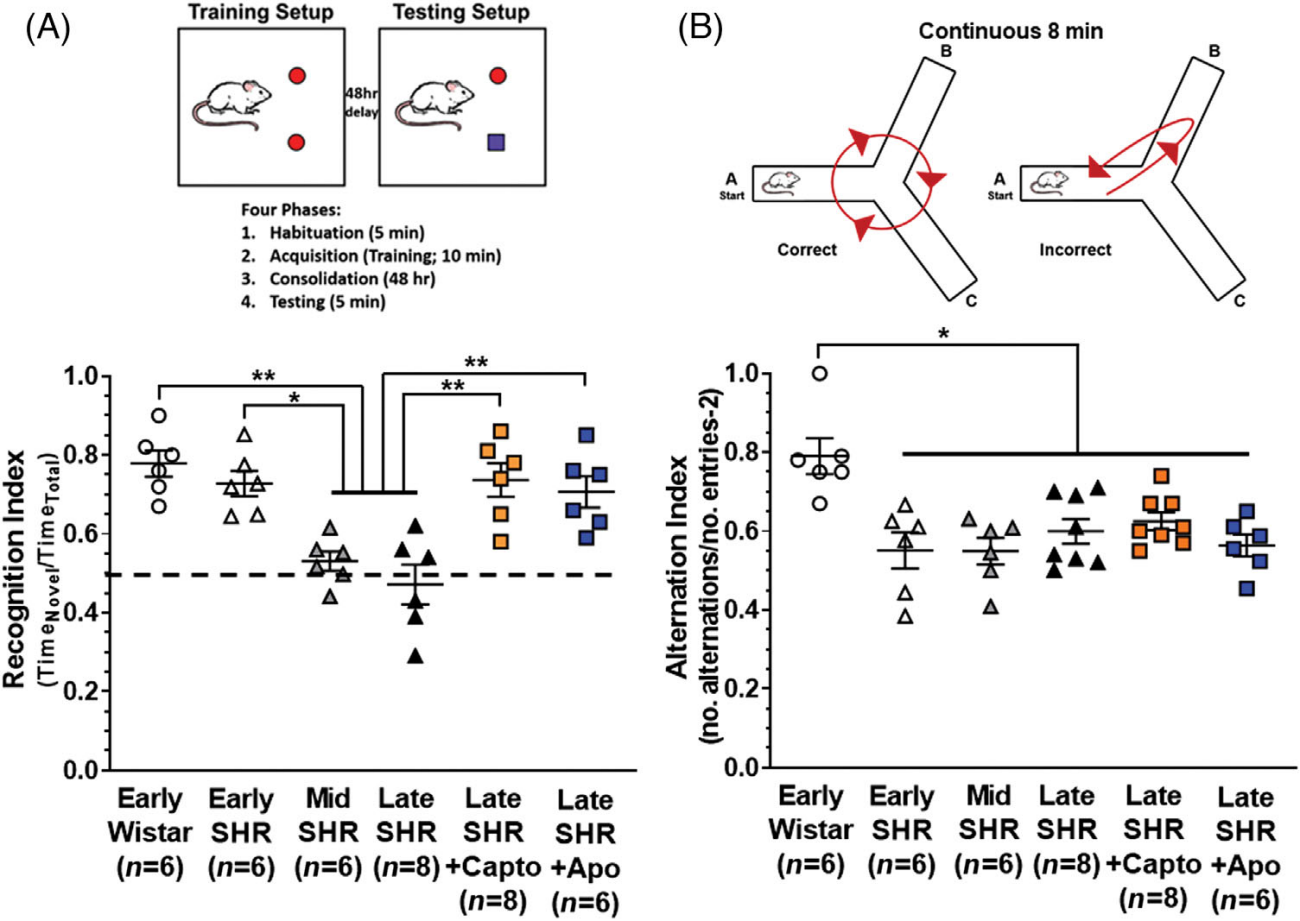
Aging during chronic hypertension differentially affected memory function and long‐term memory was selectively rescued by captopril and apocynin treatments. (A) Long‐term memory function was assessed in rats in early (4 to 5 months old), mid (8 to 9 months old), and late adulthood (14 to 15 months old) using a novel object recognition task. Long‐term memory was impaired in SHR in mid and late adulthood compared to SHR and Wistar rats in early adulthood. Captopril (+Capto) and apocynin (+Apo) treatment improved memory in SHR in late adulthood. (B) The continuous Y maze test was used to assess spatial working memory. Alternation indices were lower in SHR at all stages of adulthood and was unaffected by captopril or apocynin treatment. Data are mean ± SEM; group differences were assessed using a one‐way ANOVA test with Tukey's multiple comparison test. *p < 0.05, **p < 0.01; *n =* 6 ‐ 8/group.

To investigate the effect of age during hypertension on memory function, we performed behavioral tests in SHR at three ages: early, mid, and late adulthood. The NOR task is a test of long‐term memory that is largely hippocampal‐dependent. The NOR task utilizes rodents’ innate preference for novelty by comparing their exploration of a familiar object and a novel object. A cognitively intact rat will demonstrate a preference for the novel object when given the option to explore both objects (recognition index > 0.50). Locomotion was similar between all groups, with average speed ranging from ∼ 0.05 to 0.06 m/s in SHR that was unaffected by age. SHR in early adulthood (Early SHR) did not have deficits in long‐term memory, indicated by recognition indices that were similar to that of Wistar rats (Early Wistar; Figure 1A). However, SHR in both mid and late adulthood (Mid SHR and Late SHR) had deficits in long‐term memory, indicated by lower recognition indices (Figure 1A). Spatial working memory, which involves hippocampal‐prefrontal cortex signaling, was also investigated using a Y maze.^37^, ^49^ If the rat has intact working memory, the rat will alternate from arm A to arm B and arm B to arm C without making a repeat entry (an error), as illustrated in the top panel of Figure 1B. An alternation index was calculated as a measure of memory, with a higher index indicating better working memory function. All SHR groups had lower alternation indices compared to normotensive Wistar rats, indicating that chronic hypertension impaired spatial working memory regardless of age (Figure 1B).

#### 3.1.2 Hippocampal perfusion decreased with age during chronic hypertension

**FIGURE 2:**
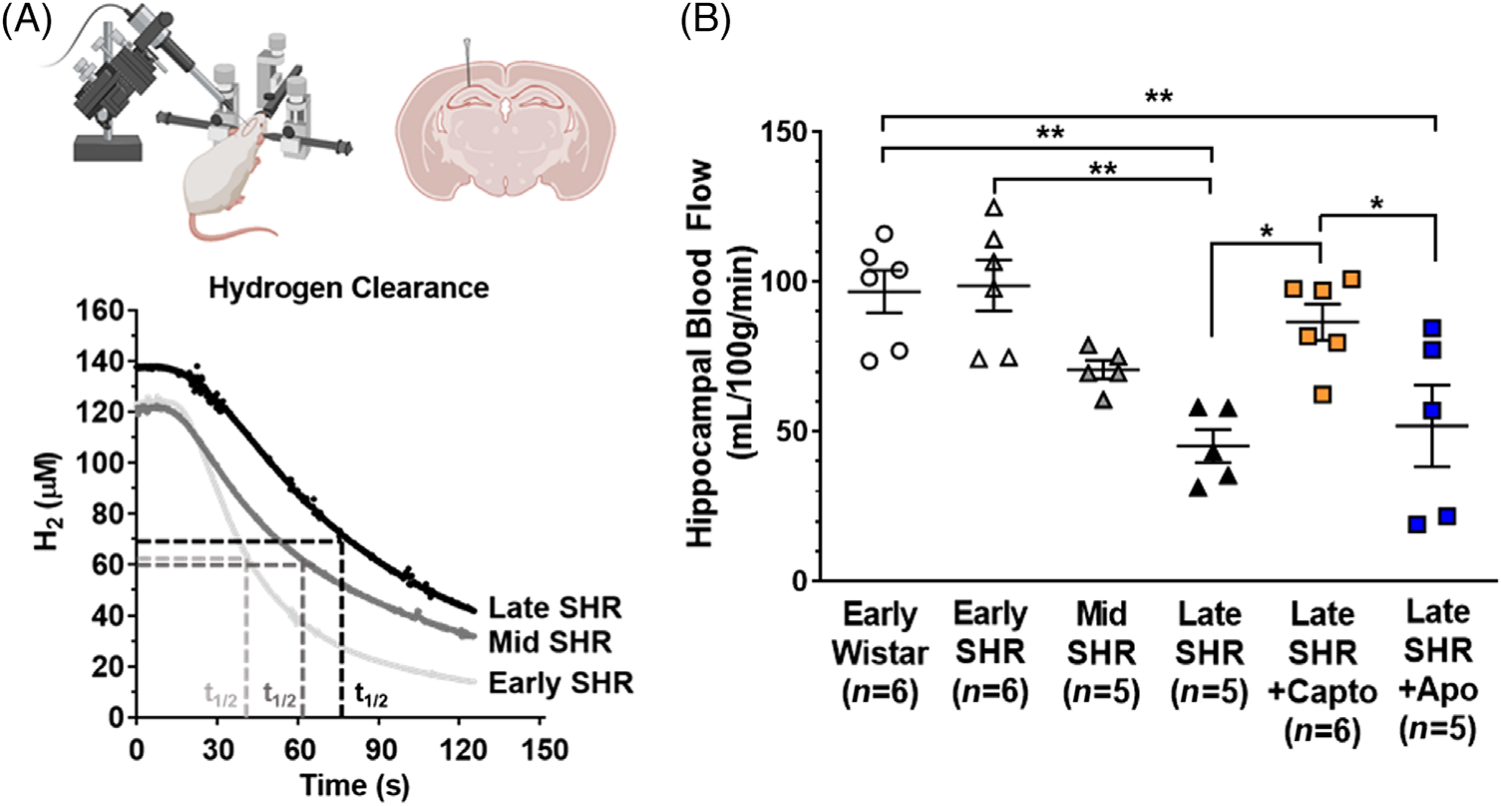
Progressive hippocampal hypoperfusion during chronic hypertension was restored by captopril treatment. (A) Schematic of hydrogen probe placement (top) and representative tracings of hydrogen clearance (bottom) are shown for each stage of adulthood studied in SHR. (B) Absolute hippocampal blood flow decreased with age in a stepwise manner in SHR and was significantly lower in SHR in late adulthood compared to SHR and Wistar rats in early adulthood. Captopril treatment (+Capto), but not apocynin (+Apo) treatment, increased hippocampal perfusion to be similar to perfusion in rats in early adulthood. Data are mean ± SEM; group differences were assessed using a one‐way ANOVA test with Tukey's multiple comparison test. *p < 0.05, **p < 0.01; *n =* 5 ‐ 6/group.

To determine whether hypoperfusion in the hippocampus might contribute to memory deficits, we measured absolute blood flow in the hippocampus in vivo using the H_2_ clearance method. Table 1 shows the physiological parameters of rats undergoing basal blood flow measurements. SHR in early, mid, and late adulthood weighed less and had significantly higher blood pressure than Wistar rats under chloral hydrate anesthesia. Blood gases and blood pH were maintained within the physiological range during hippocampal blood flow measurements. Figure 2A illustrates the stereotaxic placement of the H_2_ microsensor in the CA1 region of the hippocampus as well as representative traces of H_2_ clearance used to calculate blood flow. Hippocampal perfusion was reduced in SHR in mid‐adulthood compared to Wistar rats, although this did not reach statistical significance. By late adulthood, however, basal blood flow in the hippocampus was reduced by approximately 50% compared to SHR and Wistar rats in early adulthood (Figure 2B).

**TABLE 1:** Physiological parameters under chloral hydrate anesthesia during hydrogen clearance to measure absolute hippocampal perfusion.

	Early Wistar (*n* = 6)	Early SHR (*n* = 6)	Mid SHR (*n* = 5)	Late SHR (*n* = 5)	Late SHR + Capto (*n* = 6)	Late SHR + Apo (*n* = 5)
Body weight (g)	455 ± 19	326 ± 11^**^	364 ± 13^**^	389 ± 6^**^	372 ± 5^**^	372 ± 6^**^
PO_2 (mmHg)_	128 ± 10	118 ± 11	122 ± 9	127 ± 14	137 ± 10	118 ± 14
PCO_2 (mmHg)_	43.8 ± 1.0	37.1 ± 1.0	35.6 ± 0.9	42.0 ± 1.7	40.8 ± 2.5	36.7 ± 1.1
pH	7.42 ± 0.01	7.47 ± 0.01	7.46 ± 0.02	7.43 ± .03	7.41 ± .03	7.45 ± .01
Average BP (mmHg)	93 ± 1	147 ± 3^**^ **^#^**	138 ± 3^**^ **^#^**	136 ± 3^**^ **^#^**	97 ± 5	122 ± 14^*^

Data are mean ± SEM. ^*^ *p* < 0.05, ^**^ *p* < 0.01 versus Wistar; **^#^** *p* < 0.01 versus Late SHR+Capto by one‐way ANOVA with post hoc Tukey's test to correct for multiple comparisons.

#### 3.1.3 HAs were hyperconstricted during chronic hypertension

**FIGURE 3:**
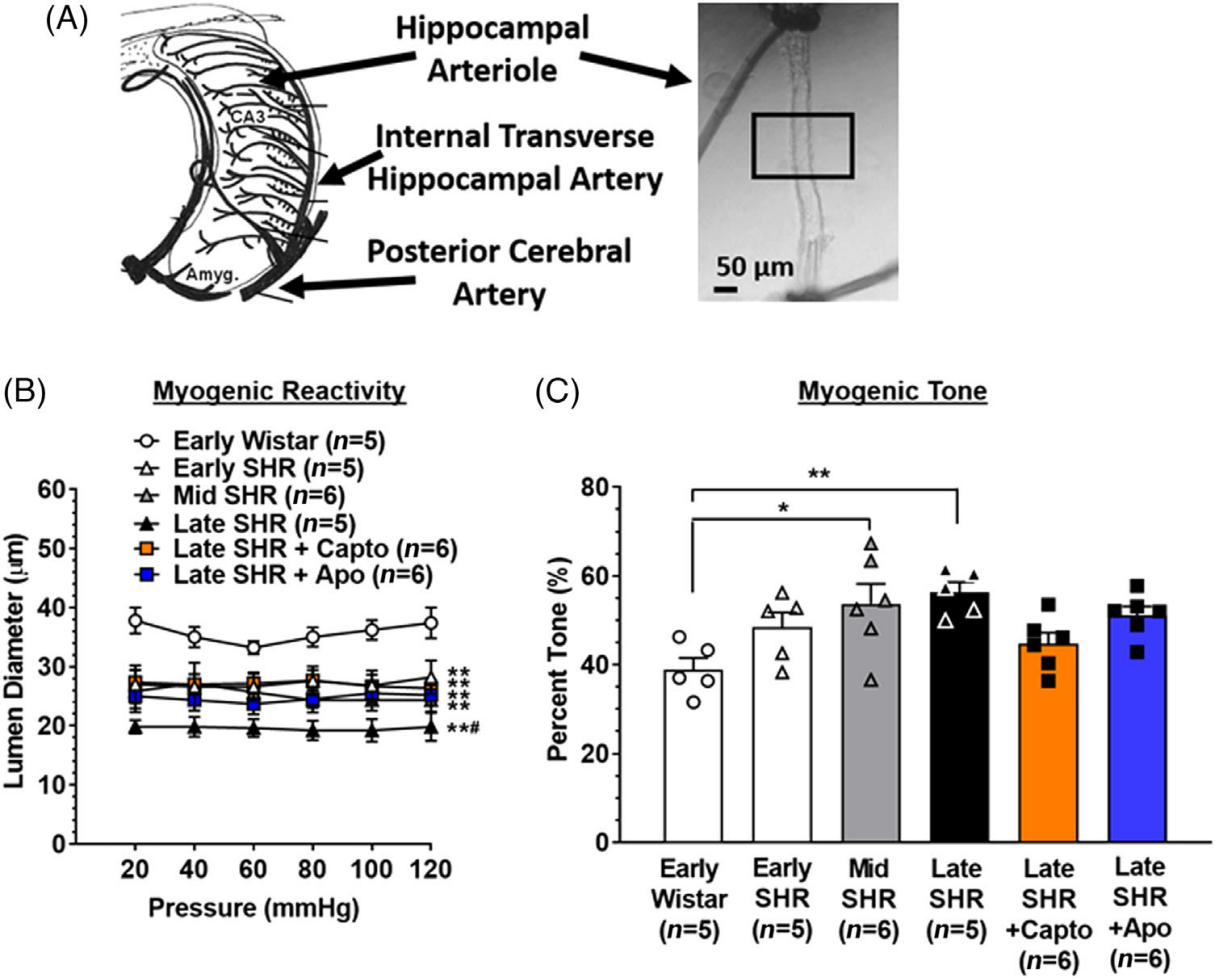
Hippocampal arterioles were hyperconstricted during chronic hypertension that increased with age. (A) Illustration of hippocampal vasculature and representative image of cannulated and pressurized hippocampal arteriole (HA) in arteriograph chamber used for pressure myography studies. (B) Lumen diameters of HAs remained relatively constant across the intravascular pressure range studied. HAs from SHR had smaller lumen diameters compared to Wistar rats regardless of age and/or treatment, with the smallest being in HAs from SHR in late adulthood. HAs from Late SHR+Capto had larger lumen diameters compared to HAs from untreated SHR in late adulthood. (C) Myogenic tone was increased in SHR in mid and late adulthood compared to arterioles from Wistar rats that was ameliorated by captopril treatment and apocynin treatment of SHR in late adulthood. Data are mean ± SEM; group differences were assessed using a one‐way ANOVA test with Tukey's multiple comparison test. *p < 0.05, **p < 0.01 versus Wistar; #*p* < 0.05 versus Late SHR+Capto; *n =* 5 ‐ 6/group.

To determine the mechanisms that contribute to hypertension‐induced reductions in basal blood flow, we studied freshly isolated HAs from separate groups of rats in vitro using pressure myography (Figure 3A). This technique allowed us to investigate the function of the arterioles responsible for perfusing the hippocampus at physiologically relevant pressures independently of neuronal and glial influences. Lumen diameters were smaller in SHR throughout adulthood at all intravascular pressures studied (20 to 120 mmHg) compared to HAs from Wistar rats (Figure 3B). Further, HAs from SHR in mid and late adulthood were hyperconstricted and had more myogenic tone at their operating intravascular pressure of 60 mmHg compared to HAs from normotensive rats (Figure 3C). However, there was no difference in tone between arterioles from Wistar rats and SHR in early adulthood, suggesting a progressive increase in HA myogenic tone with age during chronic hypertension (Figure 3C).

**FIGURE 4:**
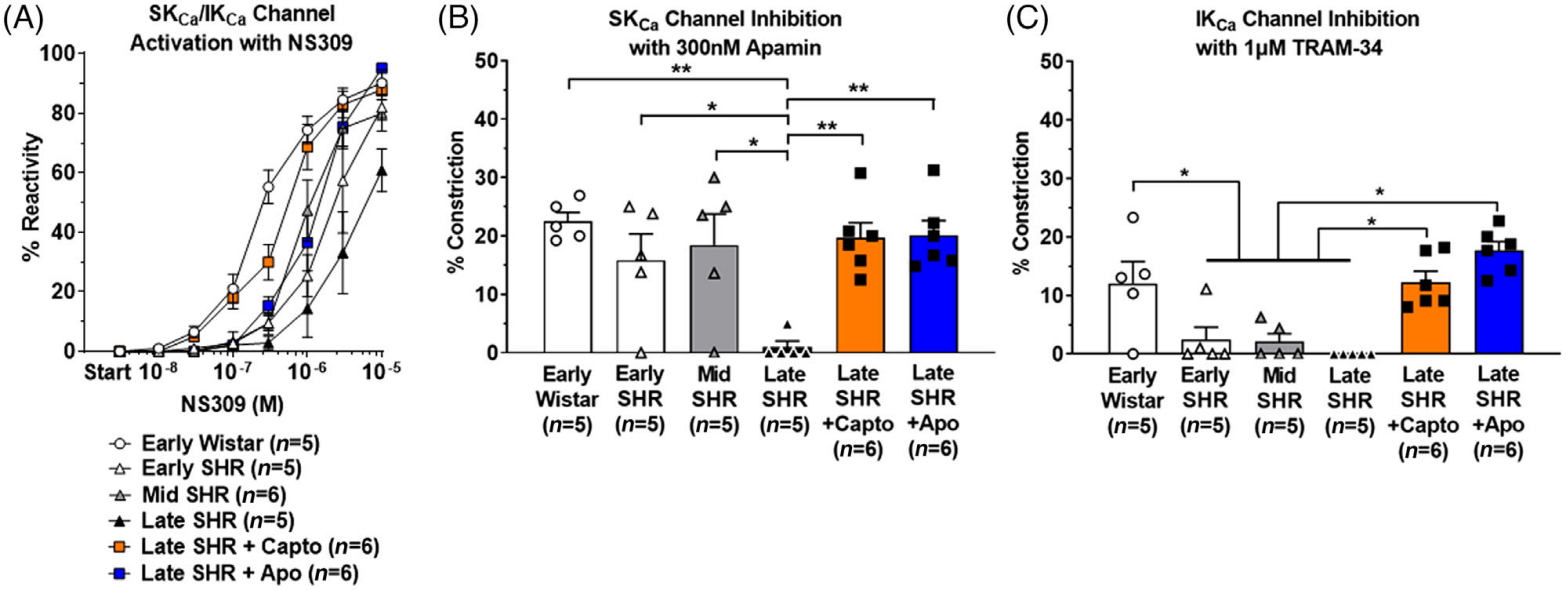
Endothelial function was impaired during chronic hypertension that worsened with age and was restored by captopril and apocynin treatment. (A) Vasoreactivity of hippocampal arterioles (HAs) from SHR in early, mid, and late adulthood and Late SHR+Apo were shifted rightward at lower doses of NS309 compared to arterioles from Wistar and Late SHR+Capto rats. However, HAs from all groups vasodilated at higher concentrations of NS309. (B) Arterioles from SHR in late adulthood demonstrated little to no response to SK_Ca_ channel inhibition that was improved in arterioles from Late SHR+Capto and Late SHR+Apo. (C) HAs from SHR at all stages of adulthood had limited vasoconstriction in response to IK_Ca_ channel inhibition compared to arterioles from Wistar rats. HAs from Late SHR+Capto and Late SHR+Apo had vasoconstrictive responses similar to those of arterioles from normotensive rats. Data are mean ± SEM; group differences were assessed using a one‐way ANOVA test with Tukey's multiple comparison test. *p < 0.05, **p < 0.01; *n =* 5 ‐ 6/group.

Chronic hypertension is widely known to damage the cerebral endothelium in the cortex; however, little is known about how hypertension and age affect the hippocampal vasculature. To investigate the potential mechanisms by which chronic hypertension caused hyperconstriction of HAs that progressed with age, we focused on endothelial function. SK_Ca_ and IK_Ca_ channels are calcium‐activated potassium channels expressed in endothelial cells that contribute to endothelium‐derived hyperpolarization (EDH) and vasodilation.^50^ HAs from all groups vasodilated in response to increasing concentrations of the SK_Ca_/IK_Ca_ channel agonist NS309 (Figure 4A). However, arterioles from SHR in late adulthood had a rightward shift in their vasoreactivity and higher EC_50_ values compared to arterioles from Wistar rats (Table 2), indicating impaired vasodilation of HAs in response to SK_Ca_/IK_Ca_ channel activation that may progress with age during chronic hypertension. To investigate basal SK_Ca_ and IK_Ca_ channel activity under healthy conditions and how chronic hypertension affected channel function throughout adulthood, we selectively and sequentially inhibited SK_Ca_ and IK_Ca_ channels pharmacologically and measured changes in lumen diameter. Figure 4B shows percent constriction of HAs in response to SK_Ca_ channel inhibition with apamin. Arterioles from normotensive Wistar rats constricted ∼20% in response to apamin, and those from SHR in early and mid‐adulthood demonstrated similar levels of vasoconstriction. However, HAs from SHR in late adulthood had blunted vasoconstriction, demonstrating little to no change in lumen diameter in response to SK_Ca_ channel inhibition (Figure 4B). In response to IK_Ca_ channel inhibition with TRAM‐34, HAs from normotensive rats constricted an additional ∼12%. HAs from SHR, however, had diminished vasoconstrictive responses throughout adulthood, with HAs from SHR in late adulthood having no response to IK_Ca_ channel inhibition (Figure 4C). These findings suggest that SK_Ca_ and IK_Ca_ channels are basally active in HAs under healthy conditions and that chronic hypertension causes progressive damage with age that likely contributes to the hyperconstriction of HAs.

**TABLE 2:** EC_50_ values of vasoreactivity of hippocampal arterioles to NS309.

Group (*n*)	EC_50_
Early Wistar (5)	0.32 ± 0.06 μM
Early SHR (5)	2.8 ± 1.0 μM
Mid SHR (6)	1.5 ± 0.42 μM
Late SHR (5)	8.2 ± 3.1 μM^**^ **^#^**
Late SHR+Capto (6)	0.65 ± 0.17 μM
Late SHR+Apo (6)	1.4 ± 0.29 μM

Data are mean ± SEM. ^**^ *p* < 0.01 versus Early Wistar; ^#^ *p* < 0.01 versus Late SHR+Capto by Kruskal‐Wallis ANOVA with post‐hoc Dunn's test.

#### 3.1.4 Chronic hypertension impaired the vasodilatory response to mediators of NVC

**FIGURE 5:**
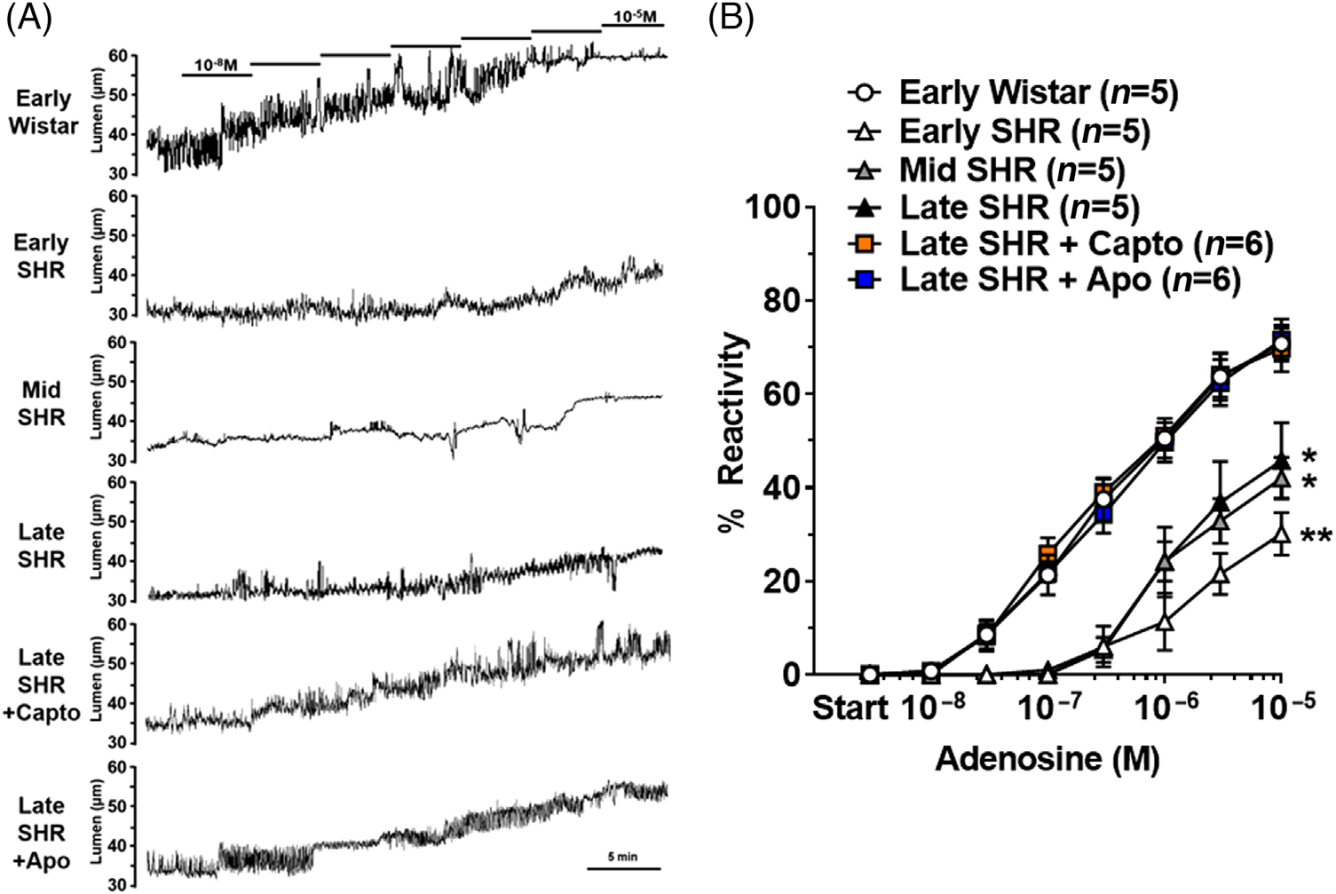
Chronic hypertension impaired the vasoreactivity of hippocampal arterioles (HAs) to adenosine. (A) Representative lumen diameter traces of HAs in response to increasing doses of adenosine. (B) Reactivity of HAs from SHR at all stages of adulthood were shifted rightward and blunted compared to HAs from Wistar rats that was improved in arterioles from Late SHR+Capto and Late SHR+Apo rats. Data are mean ± SEM; group differences were assessed using a one‐way ANOVA test with Tukey's multiple comparison test. *p < 0.05, **p < 0.01 versus Wistar, Late SHR+Capto, and Late SHR+Apo; *n =* 5 ‐ 6/group.

**FIGURE 6:**
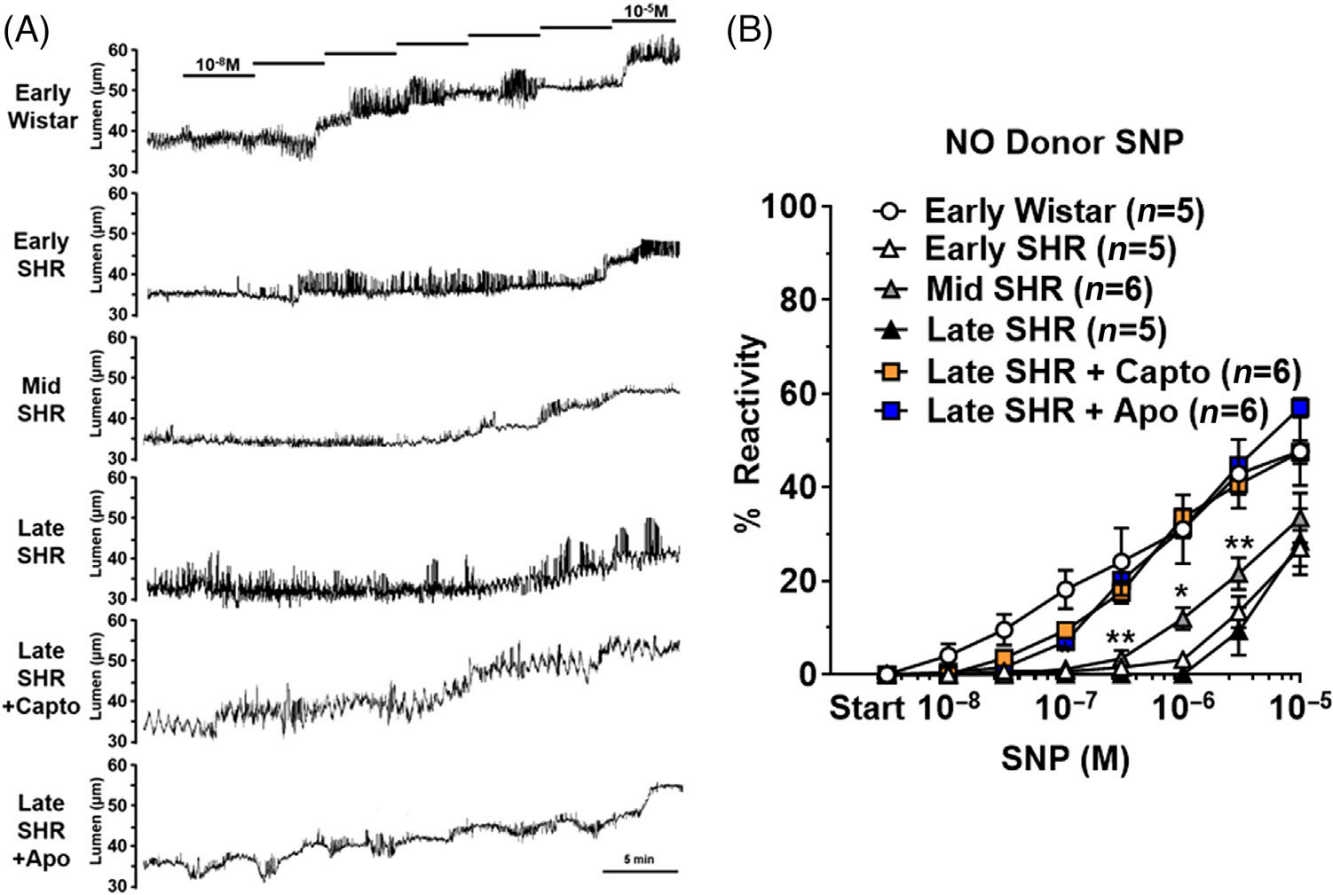
Vasoreactivity of hippocampal arterioles (HAs) to nitric oxide was impaired during chronic hypertension. (A) Representative traces of lumen diameter changes of HAs in response to increasing doses of the nitric oxide (NO) donor SNP. (B) Vasoreactivity to NO was shifted rightward and blunted in arterioles from SHR at all stages of adulthood compared to HAs from Wistar rats. Vasodilation of HAs from Late SHR+Capto and Late SHR+Apo in response to increasing concentrations of NO were similar to arterioles from Wistar rats. Data are mean ± SEM; group differences were assessed using one‐way ANOVA with Tukey's multiple comparison test. *p < 0.05, **p < 0.01 versus Wistar, Late SHR+Capto and Late SHR+Apo; *n =* 5 ‐ 6/group.

NVC is a critical aspect of maintaining appropriate blood flow to neurons in response to increases in metabolic demand. NVC in the hippocampus involves the coordinated effort of multiple cell types and the transmission of vasoactive signaling molecules, such as the metabolite adenosine and neuronal NO synthase (nNOS)‐derived NO to microvessels, to initiate local vasodilation, decrease vascular resistance, and increase local blood flow. Deficits in NVC in the cerebral cortex are present during chronic hypertension that are thought to contribute to cognitive dysfunction,^51^ but this has yet to be investigated in the hippocampus. To understand how chronic hypertension and age impact NVC in the hippocampus, we determined the critical ability of HAs to vasodilate in response to adenosine and the NO donor SNP as an indirect assessment of hippocampal NVC. Figure 5A shows representative tracings of lumen diameters in response to adenosine, where HAs from all groups vasodilated to increasing concentrations of adenosine. However, HAs from all SHR age groups had diminished reactivity compared to arterioles from Wistar rats indicating that hypertension blunted the vasodilatory response to adenosine (Figure 5B). Figure 6A shows representative tracings of lumen diameter responses to cumulative doses of SNP. Arterioles from all groups dilated in response to increasing concentrations of SNP. However, HAs from SHR in early, mid, and late adulthood had similarly impaired vasoreactivity compared to vessels from normotensive rats. Together, these data suggest that vascular aspects of NVC are impaired in the hippocampus during chronic hypertension, even in early adulthood.

#### 3.1.5 Chronic hypertension causes inward remodeling of HAs that progresses with age

Structural remodeling of the cerebrovasculature is known to occur in response to chronic hypertension such that lumen diameters are smaller and vascular walls thicker (eg, inward, hypertrophic remodeling), and this is thought to be protective of the downstream microcirculation.^52^ However, such inward remodeling limits the vasodilatory capacity of arterioles that, if severe enough or prolonged, can potentiate hypoxia and ischemia. Under fully relaxed conditions, lumen diameters of HAs from SHR in early and mid‐adulthood were smaller and had thicker walls than HAs from Wistar rats at intravascular pressures of 5 and 60 mmHg, although this did not reach statistical significance (Table S1). However, in SHR in late adulthood, HAs were significantly smaller and vascular walls thicker than arterioles from Wistar rats, demonstrating that inward, hypertrophic remodeling occurred over time during hypertension. There were no differences in cross‐sectional area or distensibility of HAs between any groups (Table S1).

### 3.2 The roles of Ang II signaling and oxidative stress in hypertension‐induced hippocampal dysfunction in late adulthood

#### 3.2.1 Captopril and apocynin improved HA function in SHR in late adulthood

We next investigated the underlying mechanisms by which chronic hypertension progressively impaired HA function, decreased hippocampal perfusion, and disrupted memory. SHR in late adulthood received chronic treatment with either the ACE inhibitor captopril or the antioxidant apocynin. As expected, captopril treatment had an anti‐hypertensive effect, with systolic/diastolic blood pressures being 124 ± 4/92 ± 3 mmHg. This was significantly lower than vehicle control SHR in late adulthood on regular drinking water (196 ± 7/147 ± 3 mmHg) and apocynin‐treated SHR (219 ± 6/172 ± 5 mmHg; *p* < 0.01), as measured by tail cuff. This reduction in blood pressure with captopril but not apocynin treatment was also apparent under chloral hydrate anesthesia (Table 1).

Structurally, captopril and apocynin treatment reversed/prevented the inward, hypertrophic remodeling of HAs from SHR in late adulthood, with lumen diameters and wall thicknesses being similar to those from SHR in early and mid‐adulthood (Table 2). Functionally, HAs from SHR in late adulthood treated with captopril and apocynin had lumen diameters that were similar in size to SHR in early and mid‐adulthood and reduced myogenic tone (Figure 3B and 3C). Vasodilatory responses to SK_Ca_ and IK_Ca_ channel activation with NS309 were improved in SHR in late adulthood that were treated with captopril or apocynin (Figure 4A). This was reflected in the leftward shift in reactivity to NS309 and EC_50_ values that were similar to those of HAs from Wistar rats (Table 2). Further, vasoconstriction of HAs from Late SHR+Capto and Late SHR+Apo rats in response to apamin (Figure 4B) and TRAM‐34 (Figure 4C) was similar to arterioles from younger SHR and normotensive controls, respectively. Finally, both captopril treatment and apocynin treatment restored the vasoreactivity of HAs to adenosine (Figure 5) and NO (Figure 6) to be similar to the reactivity of arterioles from normotensive control rats. These findings suggest Ang II‐induced oxidative stress caused endothelial dysfunction and hyperconstriction of HAs and impaired vascular contributions to NVC in the hippocampus in SHR in late adulthood.

#### 3.2.2 Improved HA function by captopril restored hippocampal perfusion and rescued memory deficit in SHR in late adulthood

To understand the relationship between HA hyperconstriction and reduced hippocampal perfusion, we measured absolute hippocampal blood flow in SHR in late adulthood that were treated with captopril or apocynin, both of which improved HA function. Interestingly, hippocampal perfusion was increased by captopril treatment in late adulthood to be similar to blood flow in SHRs in early adulthood and normotensive rats, whereas hippocampal blood flow in SHR treated with apocynin remained similarly low as in SHR in mid and late adulthood (Figure 2B). Both captopril and apocynin selectively improved hippocampal‐dependent long‐term memory function in SHR in late adulthood (Figure 1A) but had no impact on hippocampal‐prefrontal cortex‐dependent spatial working memory (Figure 1B).

## 4 DISCUSSION

Dementia is increasing in incidence as the world's population ages, affecting the majority of people over 70 years old and often involving hippocampal dysfunction.^53^, ^54^ Currently, there are no treatments for VCI, and combating the increasing incidence relies solely on preventive strategies.^55^ Chronic hypertension is known to be the primary modifiable risk factor for cerebrovascular disease and VCI. Thus, understanding how hypertension affects the vasculature of the hippocampus is critical to slowing and/or preventing vascular contributions to cognitive impairment and dementia. The majority of investigations into the mechanisms by which chronic hypertension and aging damage the cerebral circulation have been completed in the cerebral cortex and striatum. In the current study, we expanded our understanding of how chronic hypertension impairs endothelial and vascular smooth muscle function to include within the hippocampal vasculature across adulthood. Our findings showed that chronic hypertension progressively impaired endothelial function with age that caused hyperconstriction of HAs, blunted vasoreactivity to mediators of NVC, reduced hippocampal perfusion, and impaired hippocampal‐dependent memory function. Further, we showed for the first time that improving HA function during chronic hypertension via ACE inhibition restored hippocampal blood flow and selectively improved hippocampal‐dependent memory function in late adulthood. These findings not only broaden our understanding of how chronic hypertension contributes to VCI but also provide proof‐of‐principle evidence of the role of the hippocampal vasculature and maintenance of basal perfusion and activity‐dependent changes in blood flow in protecting neurocognitive health late in life.

The control of local, basal blood flow occurs at the level of precapillary arterioles and upstream penetrating arterioles, and the largest determinant of hippocampal blood flow is the lumen diameter of HAs.^56^ Thus, the structure and function of HAs are critical in maintaining basal perfusion of the hippocampus and protecting learning and memory function. Compared to other, more well‐studied cerebrovascular territories, little is known about endothelial function in the hippocampus under healthy conditions as well as pathological states known to cause endothelial dysfunction in other brain regions, such as chronic hypertenison.^20^, ^35^, ^39^ In healthy rats, SK_Ca_ and IK_Ca_ channels are basally active in parenchymal arterioles branching off the middle cerebral artery and perfusing the sensorimotor cortex and striatum, which contribute to resting cerebral blood flow.^50^, ^57^ Here, we show for the first time that SK_Ca_ and IK_Ca_ channels are basally active in the endothelium of HAs and are involved in determining hippocampal blood flow. We previously showed there to be a limited role of endothelial‐derived NO in the myogenic tone of isolated and pressurized HAs, which is in striking contrast to other cerebrovascular segments.^20^, ^35^, ^39^ Given the central role of SK_Ca_ and IK_Ca_ channels in EDH, the current findings implicate EDH as a primary tonic vasodilatory stimulus in the hippocampus. These findings expand our understanding of endothelial function in the hippocampus as it relates to myogenic tone and basal blood flow.

In the current study, chronic hypertension progressively impaired SK_Ca_/IK_Ca_ channel function with age, which contributed to increased myogenic tone, hyperconstriction of HAs, and hippocampal hypoperfusion. In SHR in late adulthood, chronic treatment with both captopril and apocynin improved endothelial function of HAs. However, only captopril improved hippocampal perfusion, although there was more variability in blood flow in SHR+Apo rats than in other groups. In addition to inhibiting production of Ang II, ACE inhibitors also inhibit the breakdown of bradykinin, a potent vasodilator in the cerebral circulation.^58^ Captopril is known to enhance bradykinin signaling and flow‐mediated vasodilation through endothelial‐dependent mechanisms, including EDH.^58^, ^59^ Therefore, the restoration of hippocampal perfusion in vivo in Late SHR+Capto could be due to the combination of decreased Ang II signaling, which improved HA SK_Ca_/IK_Ca_ channel function, and increased bradykinin signaling. Further, HAs from Late SHR+Apo still had > 50% myogenic tone, whereas HAs from Late SHR+Capto had ∼ 44% tone. Although this was not statistically significantly different, it may be biologically relevant, contributing to higher vascular resistance and persistently decreased blood flow in the hippocampus of Late SHR+Apo. The modestly higher myogenic tone in HAs from SHR+Apo may also be protective of the microcirculation in the context of sustained hypertension, as apocynin did not have anti‐hypertensive effects.

Here, we found that both captopril and apocynin treatments improved hippocampal‐dependent memory, even in the absence of restored basal hippocampal blood flow with apocynin treatment. Apocynin and captopril cross the blood‐brain barrier and therefore may directly influence neuronal health independently of actions on the vasculature, specifically by each reducing oxidative stress and neuroinflammation.^60^, ^61^, ^62^ Thus, future studies are needed, as we cannot distinguish between treatment‐specific vascular protection versus neuronally protective mechanisms in the current study. Regardless, these findings suggest that ACE inhibition is more effective at improving HA vascular function and subsequently restoring basal blood flow than treatment with an antioxidant alone. That improving HA function in SHR in late adulthood with an ACE inhibitor improved hippocampal perfusion and restored long‐term memory function, without improving working memory, suggests that hypertension‐induced HA dysfunction may play a causal role in hippocampal‐dependent memory decline and VCI.

In addition to basal blood flow, neuronal activity‐dependent changes in local perfusion (eg, NVC) is another critical aspect of cerebral hemodynamics and maintaining healthy neurocognitive function. In cortical brain regions, NVC is impaired due to arteriole dysfunction, largely through disrupted inward‐rectifying potassium channel function, and contributes to cognitive decline in models of AD. Further, NVC in the cortex is known to be impaired during chronic hypertension.^51^, ^63^, ^64^ However, little is known about how chronic hypertension affects NVC in the hippocampus, which could further contribute to memory decline with age. The primary mediator of NVC in the hippocampus has been determined to be nNOS‐derived NO that diffuses to the scarce microcirculation to cause local vasodilation.^65^, ^66^ Initiation of such vasodilation in response to hippocampal neuronal activity is a critical aspect of NVC, making the ability of HAs to vasodilate to mediators of NVC important for maintenance of neuronal health.^51^, ^56^ In the current study, the vasodilatory response of HAs to the NO donor SNP and adenosine were blunted in arterioles from hypertensive rats regardless of stage of adulthood, indicating arterioles have an impaired ability to respond to these primary, neuronally derived mediators of NVC in the hippocampus.

Another critical aspect of NVC is conducting local vasodilations to upstream arterial segments to reduce vascular resistance and increase local blood flow, that involves activity of SK_Ca_/IK_Ca_ channels.^50^, ^64^, ^67^, ^68^ As previously discussed, SK_Ca_/IK_Ca_ channel function was progressively impaired with age during chronic hypertension. Although we did not directly measure NVC in vivo in the current study and future studies are necessary to make concrete statements about NVC in the hippocampus, our findings do suggest that two critical vascular aspects of NVC are impaired in the hippocampus during chronic hypertension: the ability for neuronal activity to generate a local vasodilation and the ability of that vasodilation to be conducted upstream to increase local blood flow. Importantly, impaired vasoreactivity of HAs to adenosine and NO was present in HAs from SHR in early adulthood that demonstrated intact hippocampal‐dependent memory. These findings suggest that NVC may be impaired in the hippocampus prior to the onset of memory dysfunction and that HA dysfunction may play a causal role in hypertension‐induced memory decline and VCI. Further, improvement of HA reactivity to NO and adenosine in SHR in late adulthood with captopril or apocynin treatment suggests these treatments improved hippocampal NVC that could contribute to the rescue of memory function seen in these groups. Although late adulthood was the primary focus for therapeutic intervention in the current study, future studies are necessary to investigate whether treatment initiated in early adulthood, prior to a decline in memory function, could improve aspects of NVC and prevent/slow hypertension‐induced memory dysfunction.

Chronic hypertension is known to accelerate age‐related cognitive decline, including VCI and vascular dementia. The findings of this study provide new insight into how chronic hypertension and concomitant aging impact hippocampal function and suggest a causal role of HAs in hypertension and age‐related memory decline. Cognitive impairment and dementia are leading contributors to disability and are increasing at an alarming rate, likely due to the aging of the population and absence of effective treatments.^69^ The hippocampal vasculature is gaining momentum as a critical player in maintaining neurocognitive health and contributing to memory decline associated with healthy aging as well as hypertension‐related cerebrovascular disease and VCI.^19^, ^20^ It is well established that anti‐hypertensive medications that target the renin‐angiotensin system (eg, ACE inhibitors, Ang II receptor blockers) are associated with decreased progression to probable dementia, including AD, as well as in rodent models of chronic hypertension.^70^, ^71^, ^72^ The findings in the current study suggest that the hippocampal vasculature may be a previously unrecognized therapeutic target and represents a novel mechanism by which ACE inhibition may slow memory decline during chronic hypertension.

## CONFLICTS OF INTEREST STATEMENT

The authors have no competing interests to declare (supporting information).

## CONSENT STATEMENT

Consent was not necessary.

## Supporting information

Supporting Information

Supporting Information
