# Supplementary material for: Angiotensin II‐mediated hippocampal hypoperfusion and vascular dysfunction contribute to vascular cognitive impairment in aged hypertensive rats
Source: Alzheimers Dement. 2023 Oct 10;20(2):890–903. doi: 10.1002/alz.13491 (PMC10917018; doi:10.1002/alz.13491)
Supplement: Supplementary file 1 — Supporting Information [file ALZ-20-890-s002.docx]

**SUPPLEMENTARY MATERIAL**

**Angiotensin II-mediated hippocampal hypoperfusion and vascular dysfunction contributes to vascular cognitive impairment in aged hypertensive rats**

Olivia Gannon, Sarah M. Tremble, Conor McGinn, Ruby Guth, Nadia Scoppettone, Ryan Hunt, Kirtika Prakash, and Abbie C. Johnson

**Supplementary Table 1. Structural properties of hippocampal arterioles under fully relaxed conditions.**

|  | **Early Wistar (n=5)** | **Early SHR (n=5)** | **Mid SHR (n=5)** | **Late SHR (n=5)** | **Late SHR**  **+ Capto (n=6)** | **Late SHR**  **+ Apo (n=6)** |
| --- | --- | --- | --- | --- | --- | --- |
| Lumen_5_ (μm) | 38.6±1.2 | 34.4±1.7 | 33.8±2.5 | 28.0±1.4** | 34.0±3.0 | 32.5±0.6 |
| Lumen_60_ (μm) | 61.4±2.4 | 51.4±2.6 | 50.3±3.9 | 42.6±2.9** | 49.3±3.8* | 47.3±0.8* |
| Wall_5_ (μm) | 7.2±0.4 | 8.4±0.5 | 8.8±0.2 | 9.0±0.3****^#^** | 7.0±0.0 | 7.3±0.2 |
| Wall_60_ (μm) | 4.8±0.4 | 6.4±0.5 | 6.2±0.2 | 6.6±0.5****^#^** | 4.7±0.2 | 5.0±0.3 |
| CSA_5_ (μm^2^) | 1037±67 | 1135±108 | 1183±71 | 1049±68 | 902±67 | 918±31 |
| CSA_60_ (μm^2^) | 1007±114 | 1173±147 | 1090±65 | 1038±148 | 793±73 | 824±49 |
| % Distensibility_60_ | 59.0±3.1 | 49.6±3.6 | 49.5±4.1 | 51.8±3.9 | 46.3±3.3 | 45.7±1.3 |

Data are mean ± SEM. *p<0.05, **p<0.01 vs. Wistar; **^#^**p<0.05 vs. Late SHR+Capto and Late SHR + Apo by one-way ANOVA with post-hoc Tukey’s test to correct for multiple comparisons.
